# Age Limitations for Presidential Candidates? Exploring Student Perspectives Using Classroom Debates

**DOI:** 10.1093/geroni/igab046.741

**Published:** 2021-12-17

**Authors:** Jason Garbarino

**Affiliations:** University of Vermont, Burlington, Vermont, United States

## Abstract

Ahead of the 2020 Presidential Election, Donald Trump (age 73) and his primary opponent, Joseph Biden (age 76) received extensive criticism regarding the aptness of their candidacies based upon their current ages. While the United States Constitution requires candidates to have “attained the age of thirty-five years”, no age cap for presidential candidates exists. In response to timely public discussion, undergraduate interprofessional gerontology students worked in assigned groups to prepare to debate either in favor of, or in opposition to a constitutional amendment capping the age of presidential candidates. Following classroom debates, course faculty moderated in-depth conversation examining cogent arguments made throughout the debates. After attending this session, participants will understand the logistics of planning in-class debates, moderating post-debate student discussions, and evaluation methods of student debate performance and on a corresponding reflective writing assignment. Student and faculty takeaways and prospective classroom debate ideas will be provided.

